# Gastric Antral Diverticula: A Rare Diverticula with a Unique Presentation

**DOI:** 10.1155/2021/6623183

**Published:** 2021-03-26

**Authors:** Pratishtha Singh, Kathleen Raynor, Chadley Froes

**Affiliations:** Department of Internal Medicine, Grand Strand Medical Center, Myrtle Beach, SC, USA

## Abstract

Gastric diverticula are the least common gastrointestinal diverticula. Patients can be diagnosed incidentally on EGD or present with variable symptoms such as abdominal fullness, anorexia, and perforation. Gastric diverticula can be acquired from malignancy, peptic ulcer disease, or prior surgery or be congenital. Treatment varies based on symptomatology ranging from conservative medical management with proton pump inhibitors to surgical treatment with open or laparoscopic resection. We present a case of a 73-year-old female with acquired gastric diverticulum presenting as a gastric outlet obstruction who was successfully treated with conservative medical therapy.

## 1. Case Report

A 73-year-old female with a history of peptic ulcer disease (PUD) presented with intractable nausea, vomiting, and epigastric pain. Her PUD was initially diagnosed in the 1980's as an ulcer initiated by an upper gastrointestinal (GI) series and successfully treated with omeprazole. There was no reported treatment for *H. pylori* infection and esophagogastroduodenoscopy (EGD) at that time.

Five months prior to presentation, she was seen by her gastroenterologist for altered bowel habit and new onset diarrhea with scant blood. She underwent a colonoscopy one month ago which only revealed mild diverticulosis. On admission, she reported having indigestion ongoing for several months and described it as bloating, early satiety, and anorexia. Two weeks prior to presentation, she developed abdominal distention with several episodes of severe nausea and bilious vomiting. The patient admitted to using aspirin/paracetamol/caffeine almost daily for the last 4 months for headaches. She reported stopping it for her colonoscopy ten days prior to admission.

**Figure 1:**
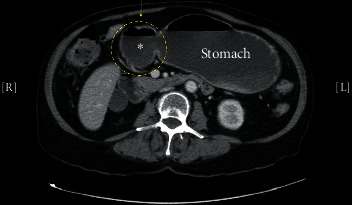
Initial abdomen computed tomography scan showing nonspecific fluid distension of the stomach with possible diverticulum (circle).

In the ED, her vital signs were normal. A nasogastric tube was placed (with bilious drainage). Her labs on admission were remarkable for leukocytosis to 17,700/mm^3^, with a normal basic metabolic panel, lipase level, and liver function panel. Abdominal X-ray was unremarkable, and computed tomography (CT) of the abdomen (Figure 1) showed nonspecific fluid distention of the stomach and duodenum.

**Figure 2:**
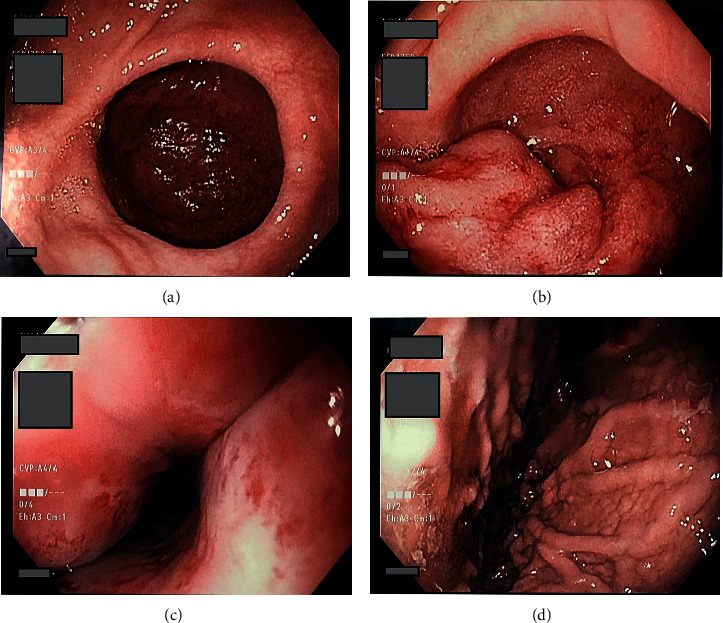
Endoscopic view of the gastric diverticulum, located in the antrum. (a) Bottom left-large antral diverticula. (b) Top left-inflammation within the diverticulum. (c) Bottom right-erosive esophagitis. (d) Top right-retroflex look at fundus/cardia.

**Figure 3:**
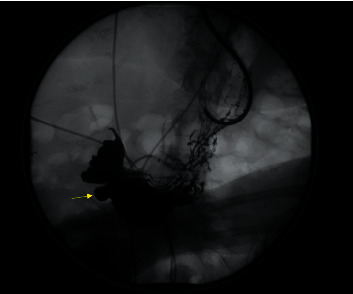
Upper gastrointestinal contrast radiographic study. The patient was discovered to have a distal antral diverticulum of the under surface of the gastric body (arrow).

CA-19 and CEA were drawn for diagnostic assessment of malignancy and were noted to be normal. She was initially admitted for a possible gastric outlet obstruction as seen on CT. The patient underwent an EGD (Figure 2) which revealed grade D erosive esophagitis and a large diverticulum in the prepyloric region causing a marked deformity of the pylorus. The diverticulum was estimated to be greater than 10 cm, wide mouth, and ulcerated with no stigmata of active bleeding noted. Biopsies were obtained at this time to rule out malignancy and test for *H. pylori*. Her magnetic resonance cholangiopancreatography (MRCP) was unremarkable for any pancreatic or duodenal abnormalities. An upper GI series (Figure 3) showed a distal antral diverticulum. Hepatobiliary surgery service was consulted, and options for treatment were discussed including minimally invasive treatment options and surgery. However, as patient was at high risk for perforation, conservative management was started initially with twice daily PPIs for several weeks and bowel rest. She continued to improve with resolution of her presenting symptoms. She was able to tolerate a full diet and was discharged on hospital day four.

Two weeks later, on outpatient follow-up, an abdominal CT showed resolution of the gastric distention. Repeat EGD at three months showed diverticulum in the prepyloric region without any evidence of obstruction. Continued deformity of the prepyloric area and scarring from her previous PUD was noted. Patients' initial biopsies were negative for malignancy and positive for *H. pylori*, and she was started on therapy with bismuth subcitrate potassium, metronidazole, and tetracycline (used due to known allergies to several antibiotics) along with PPI. A follow-up breath test to check for eradication was negative.

## 2. Discussion

Gastric diverticula (GD) are rare congenital or acquired outpouchings of the gastric mucosa. Most are small and solitary 1–6 cm projections that go largely unnoticed, although cases of large 20 cm diverticula have been described with significant symptomatology. GD represent the least common variety of diverticular disease, with a prevalence ranging from 0.01%–0.11% on EGD, with 0.02% found on autopsy [1]. Males and females are equally affected, and while the majority of patients are 40–60 years of age at the time of diagnosis, pediatric cases have been described in the literature [2].

Congenital GD account for upwards of 70% of all cases and classically arise on the posterior aspect of the lesser curvature, near the gastroesophageal junction [1]. They are thought to occur when a portion of the gastric fundus herniates through the dorsal mesentery, prior to embryonic fusion with the left posterior body wall [2]. While they initially lie superior to the pancreas, peristaltic stretching during development projects them posteriorly and into the retroperitoneal space.

Acquired GD are less common pseudodiverticula that lack muscular layers and are generally found near the gastric antrum [3]. They are classically associated with comorbid malignancy, peptic ulcer disease, intestinal adhesions, or as a complication of bariatric surgery [4]. Acquired diverticula can be further subclassified into traction type and pulsation type, depending on the underlying pathogenesis [5]. Traction diverticula are the product of perigastric adhesions, which can form either as a complication of prior surgery or secondary to underlying peptic ulcer disease, pancreatitis, GERD, or malignancy. Pulsation diverticula are related to states of increased intraluminal pressure, often in the context of pregnancy, obesity, or chronic coughing [1, 6].

Given that symptoms can be extremely variable, a high index of suspicion is necessary to make the diagnosis. Most patients are largely asymptomatic and are diagnosed only by incidental findings on EGD. When present, clinical symptoms are nonspecific, ranging from abdominal fullness and anorexia to frank gastrointestinal bleed or perforation [1, 4, 7]. Most commonly, GD presents as nonspecific epigastric pain with anorexia [7].

GD was historically diagnosed using barium swallow studies, and while contrasted CT may provide some diagnostic utility, quantitative comparison of imaging sensitivity has not yet been established. Direct visualization via EGD appears to offer the greatest utility, although a combination of imaging modalities is typically required, given its low incidence and nonspecific symptomatology [1, 7]. In our case, GD was unrecognized (as nonspecific fluid distention) on initial abdominal CT and was only later appreciated during subsequent EGD with confirmatory upper GI series. This reflects the significance of EGD and contrasted swallow studies as the gold standard for the diagnosis of GD, per prior radiologic reviews [8].

Treatment tends to be proportional to clinical presentation, with asymptomatic diverticula often requiring no intervention [1]. Mildly symptomatic cases may benefit from medical management with either H2-blockers or proton pump inhibitors. Large diverticula or those with severe symptoms are generally amenable to open or laparoscopic resection. In addition to relieving clinical symptoms, surgical management may also be pursued to prevent complications such as perforation, hemorrhage, and malignancy [9].

Although perforation and gastric tumors are both rare complications, their existence reaffirms the importance of prompt recognition and treatment of gastric diverticulum, especially in symptomatic cases. Our case represents an uncommon presentation of GD masquerading as gastric outlet obstruction, reinforcing the importance of including it in the differential when approaching cases with nonspecific abdominal findings unattributable to more common gastric pathology.

## Data Availability

The data used to support the findings of this study are available from the corresponding author upon request.
